# WSES SM (World Society of Emergency Surgery Summer Meeting) highlights, education in emergency surgery

**DOI:** 10.1186/1749-7922-3-35

**Published:** 2008-12-16

**Authors:** Michael D Kelly

**Affiliations:** 1 Department of Upper GI Surgery, Frenchay Hospital, Bristol, BS16 1LE, UK

## 

This is the text of a talk given at the WSES Meeting in July 2008, Bologna, Italy.

I was interested that I was asked to give a talk on education rather than training in emergency surgery. Training implies to make someone proficient at a particular task by practice or repetition whereas education means to become knowledgeable about a topic. Emergency surgery encompasses so many possible scenarios that I think we have to take a pragmatic view and aim to educate the trainees in the principles.

I think everyone realises that increasing specialisation has brought problems with emergency and general surgery. There are multiple reasons for these changes including the sheer volume of medical knowledge, new technologies, and the general acceptance that practising outside of your special area of expertise is unacceptable. On top of this is the push by doctors for a better lifestyle, the demands of patients and the medicolegal environment.

There have been recent documents sent out by the various surgical colleges highlighting the problems with emergency surgery. I know that in the UK there has been significant political pressure on things such as elective surgery waiting times but non-trauma emergency surgery does not really grab the headlines and does not seem to be a priority for government authorities or even surgeons themselves.

So what is an emergency surgeon? Well I think that he or she must have the following attributes:

Good clinical judgement

Good practical skills

Good communication skills

Be decisive

React well to stressful situations

And finally they must have a sound knowledge of the topic.

It is my opinion that some of these attributes are difficult to teach and the selection process is critical so as to select appropriate trainees.

A broad knowledge base is the obviously the cornerstone of effective practice. However the amount of information is now so vast that a surgeon cannot know it all and does need to constantly revise topics. Emergency surgery is slightly different from other medical fields as there is a need for immediate decisions and so if the surgeon needs to look things up then he or she needs to get to the information fast.

In Commonwealth countries the Text book of Emergency Surgery by Hamilton Bailey was considered a must read. Now this book, the fifth edition published in 1945 in Bristol, was about 2 kilos, which is about the weight of a modern laptop (fig 1). Newer editions are still being produced with editors and a multi-author format. What was it that captured people's imagination and made this book such a success? I think it was the style of the writing and the numerous colour photographs.

**Figure 1:**
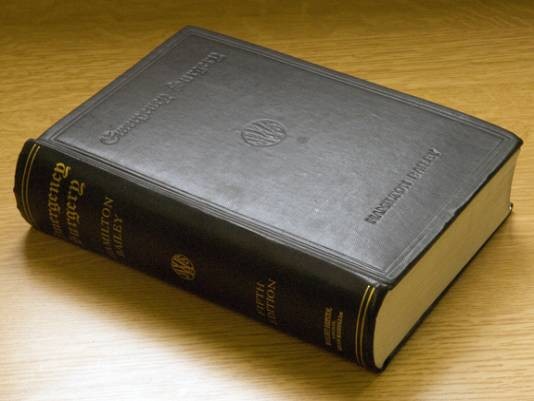
Emergency surgery by Hamilton Bailey, 5^th ^Edtn.

I think Hamilton Bailey realised how important illustrations were and how a picture can tell a thousand words. It was concise yet briefly summarised the literature on the topics. There were also anecdotal case scenarios interspersed within the text. But most importantly it conveyed a sense of drama that got the reader hooked on the subjects. It is interesting to me that over half of the most accessed articles in World Journal of Emergency Surgery are case reports often with numerous photographs.

Actually Hamilton Bailey while a busy surgeon wrote two other books (Bailey and Love's Short Practice of Surgery and Demonstrations of Physical Signs in General Surgery), which have become classics and are still in print in revised forms. This was quite an achievement but his life became a bit tragic and he did end up in a psychiatric institution before making a recovery. So maybe that is a lesson to us all not to work too hard.

Today the sheer volume of information and technical expertise has become so vast that it is virtually impossible to write a textbook covering adult and paediatric, emergency and trauma surgery. I know the following two talks are on the topic of emergency surgery books and manuals so I will be interested to hear some expert opinion on this.

Journals are important but the days of waiting for a monthly surgical journal to be delivered by the postman and then reading it from cover to cover are long gone.

Monographs or journal supplements summarising current information on a condition are useful and this is an example of one from WW2 to update surgeons on arterial injuries (Fig 2).

**Figure 2:**
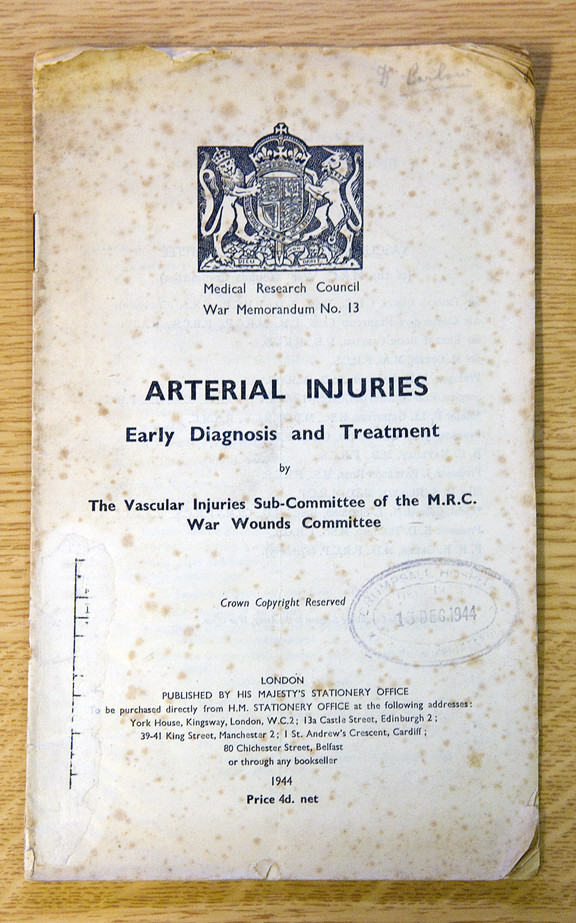
Monograph from the second world war to update surgeons on arterial injuries.

Recent advances in information technology and communication have changed forever the practice of emergency surgery in the 21^st ^century. Although a broad knowledge base is important it is just as important today to have the skills to be able to access knowledge on the internet and to have systems in place to have subspecialty experts available to give advice.

However simply being able to talk to a subspecialty colleague on the phone is not always a solution. I think we have all had the experience of asking for advice from a colleague on the telephone and being entirely happy with doing what they advise. The man or woman on the spot in emergency situations has to make the decision based on their judgement

So I think there will always be a role for the well trained general surgeon with a broad knowledge base that can be applied to patients with a wide range of problems. There are certain basic principles that once learnt are generally applicable to the management of surgical emergencies be they traumatic or nontraumatic. These principles are best learnt by dealing with major traumatic injuries and serious abdominal conditions with shock and sepsis.

Classically training in surgery, which is a skills based craft, followed the apprentice model whereby the trainee learnt by watching a master. There was no formalised education and learning was on the job. There were long hours with a high volume of cases and it could be argued that this system worked very well. For a variety of reasons this system is no longer acceptable and proper systems are in place to train and assess surgeons. There have been radical changes in surgical education with emphasis on teaching rather than learning by observation and there is now continual assessment of competency. There are validated models to assess technical proficiency at tasks such as suturing and virtual training is well established especially for laparoscopic surgery. Less than half of emergency admissions require operations so it could be argued that technical proficiency, although a bonus, is not the most important attribute [1].

I thought I would try to introduce a little bit of science into this rambling talk. A recent paper by Ledwidge and colleagues from England discussed what I believe is the main impediment to educating emergency surgeons: lack of continuity of care [2].

The European Working Time Directive (EWTD) and other reforms have meant that surgical trainees carry out fewer operations in the United Kingdom [2,3]. Laparotomies carried out by advanced surgical trainees in a general hospital fell from 54% of total laparotomies in 1995 to 18% in 2002. The corresponding figures for appendectomy by more junior trainees were 60% to 40% [2,3].

Traditionally a trainee would see the patient initially, make a diagnosis and arrange tests if appropriate. For cases needing an operation, he or she would arrange preoperative care, consent the patient, do the operation, manage the postoperative care and see the patient in the outpatient clinic for final follow-up. This was a great way to learn about emergency surgery and gave a high level of professional satisfaction. Tight regulation of working times do not allow this type of continuity of care as we knew it when we were training.

Ledwidge and colleagues found that only 26% of emergency patients were operated on by the person who assessed them initially [2]. Similarly, they found of patients managed conservatively, only 42% were reviewed during the admission by the surgeon who assessed them initially [2]. Overall, only 11% of patients who underwent an operation had the same assessor for all stages of care and 29% of those who were conservatively managed did [2]. Looking at the results of Ledwidge's study I find it hard to imagine how these trainees were not interested in what happened with the patients they had seen. Presumably they were so busy that they became tired and disinterested.

There are other similar studies from North America. One study from an academic vascular unit found that of 159 patients undergoing a vascular procedure (emergency and elective) there was a complete hospital care rate of 57% [4]. So it may be that there is more continuity of care in America. Certainly it would seem from conversations I have had with colleagues that the EWTD is not constraining training in surgery equally in all countries in the European Union.

The final word on continuity of care should really be left to the patients. I was very pleased to see in the article from Ledwidge and colleagues a reference for this showing that 61% of patients would prefer to be seen by a junior doctor they knew accepting that the doctor may be tired and consequently more likely to make mistakes [2,5].

Inspirational teachers are enthusiastic and passionate about their subject. I think we can all recall an inspirational surgeon who made us want to take up surgery. It is up to us to set high standards and bring out the best in our trainees. We need to stimulate them to follow up on their cases within the constraints of the EWTD. It is lucky that we have such an exciting topic; emergency surgery is full of drama and excitement. That is one reason why the TV program ER became such a success because the subject is so inherently interesting. We need to inspire our young doctors to do emergency surgery properly rather than to watch it on TV!

I believe there is a great opportunity for the WSES to take a lead in modern education in emergency surgery. The time is right for an international society that knows no national boundaries. Our society has a catchy name and logo. It could be a channel to show new techniques and in my field of laparoscopic surgery online videos of emergency abdominal operations would be very helpful as they could be accessed in an emergency. I believe edited and unedited videos should be available as unedited video is often of more help to show the steps in a particular operation.

Similarly WJES has a very marketable brand name and importantly is an open access online Journal. This allows a large number of high quality colour photos to accompany the text and allows the references to be linked. For the Third world where trauma and emergency surgery are a huge burden, the Society and the Journal could potentially become extremely important for education.

Challenges for the Society and the Journal are language and cultural differences across countries. To restrict the Journal to english might exclude many people from submitting their work or accessing the website. For example, it may be more useful and appropriate to add the French language for African countries and Spanish for South America.

So in summary I believe we have to take a pragmatic approach to educating a surgeon to manage emergency cases. There are so many conditions and scenarios that we need to educate trainees on the general principles. Once these principles have been learnt most emergency cases can be managed satisfactorily. Education in emergency surgery takes place on the job and we need to encourage our trainees to spend time in the emergency and operating rooms.

## Competing interests

The author declares that he has no competing interests.
